# Gastrointestinal Cell Lines Form Polarized Epithelia with an Adherent Mucus Layer when Cultured in Semi-Wet Interfaces with Mechanical Stimulation

**DOI:** 10.1371/journal.pone.0068761

**Published:** 2013-07-15

**Authors:** Nazanin Navabi, Michael A. McGuckin, Sara K. Lindén

**Affiliations:** 1 Department of Medical Biochemistry and Cell Biology, Institute of Biomedicine, University of Gothenburg, Gothenburg, Sweden; 2 Immunity, Infection and Inflammation Program, Mater Medical Research Institute and the University of Queensland School of Biomedical Sciences, Translational Research Institute, Woolloongabba, Australia; Institut Pasteur de Lille, France

## Abstract

Mucin glycoproteins are secreted in large quantities by mucosal epithelia and cell surface mucins are a prominent feature of the glycocalyx of all mucosal epithelia. Currently, studies investigating the gastrointestinal mucosal barrier use either animal experiments or non-*in vivo* like cell cultures. Many pathogens cause different pathology in mice compared to humans and the *in vitro* cell cultures used are suboptimal because they are very different from an *in vivo* mucosal surface, are often not polarized, lack important components of the glycocalyx, and often lack the mucus layer. Although gastrointestinal cell lines exist that produce mucins or polarize, human cell line models that reproducibly create the combination of a polarized epithelial cell layer, functional tight junctions and an adherent mucus layer have been missing until now. We trialed a range of treatments to induce polarization, 3D-organization, tight junctions, mucin production, mucus secretion, and formation of an adherent mucus layer that can be carried out using standard equipment. These treatments were tested on cell lines of intestinal (Caco-2, LS513, HT29, T84, LS174T, HT29 MTX-P8 and HT29 MTX-E12) and gastric (MKN7, MKN45, AGS, NCI-N87 and its hTERT Clone5 and Clone6) origins using Ussing chamber methodology and (immuno)histology. Semi-wet interface culture in combination with mechanical stimulation and DAPT caused HT29 MTX-P8, HT29 MTX-E12 and LS513 cells to polarize, form functional tight junctions, a three-dimensional architecture resembling colonic crypts, and produce an adherent mucus layer. Caco-2 and T84 cells also polarized, formed functional tight junctions and produced a thin adherent mucus layer after this treatment, but with less consistency. In conclusion, culture methods affect cell lines differently, and testing a matrix of methods vs. cell lines may be important to develop better *in vitro* models. The methods developed herein create *in vitro* mucosal surfaces suitable for studies of host-pathogen interactions at the mucosal surface.

## Introduction

The mucosal surfaces of the gastrointestinal tract are the first site where invading pathogens encounter the host. Gastrointestinal epithelial cells secrete many defensive compounds into the mucosal fluid, both constitutively and in response to microbes. Among them, mucin glycoproteins secreted by mucus producing cells in the epithelium or submucosal glands produce a layer of viscous mucus which acts as a lubricant, physical barrier and a trap for pathogens, as well as creating a matrix for other antimicrobial molecules [1], [2]. The thickness of mucus layer is variable along the gastrointestinal tract and is thickest in the colon and thinnest in the jejunum [1]. In the murine colon, the mucus layer is built up by two layers: an inner layer that is sterile and an outer layer that is the habitat of the commensal flora [3]. In the small intestine, the mucus layer is thinner and upon removal of the loose mucus gel, only a very thin discontinuous mucus layer remain [1], [4]. MUC2 is the major component of the intestinal mucus layer. In the healthy human stomach the MUC5AC and MUC6 mucins are secreted and together they produce a laminated mucus layer in which the majority of layers are MUC5AC [5].

Underneath this mucus layer, the apical surface of mucosal epithelial cells is covered by transmembrane glycoproteins known as cell surface mucins [6]. In the stomach MUC1 is the main cell surface mucin, whereas MUC3, MUC4, MUC12, MUC13 and MUC17 are produced in the intestine [7]. These membrane-bound mucins act as a barrier and most likely also as a sensor to changes in the surrounding milieu (such as pH, ionic composition, pathogens), which may result in induction of a reporting signal from their cytoplasmic tails [8].

Encounter with microbial products can increase production of mucins by mucus producing cells [9], [10], and can result in a massive discharge of mucin. This stimulation occurs directly via local release of bioactive factors as well as indirectly via activation of the host immune cells, resulting in release of inflammatory cytokines. The outcome is a rapid discharge of stored mucin secretory granules, accompanied by a thousand fold expansion in volume upon hydration to form mucus [11]. The expression of virulence factors, adherence to epithelial cells and proliferation of mucosal pathogens such as *Helicobacter pylori* and *Campylobacter jejuni,* as well as host cell cytokine signaling in response to infection, have been shown to be regulated by interactions with mucins [12]–[16].

To investigate the mechanisms by which microbes adhere, invade and signal to the host, together with the mammalian cell response, different models including cancer cell-lines, organ cultures of explanted tissue and animals have been used. Despite the fact that the mucins expressed by the most commonly used animals such as rats and mice are orthologous to human mucins, there are important differences in glycosylation. This distinction might be the reason underlying some of the differences in infectivity/pathogenicity of different microbial pathogens, as the bacteria often adhere to the host via lectin type adhesion [17]. One example is the difference in response to *H. pylori* infection between human and other animals. This gram-negative bacterium can induce peptic ulcer disease, chronic gastritis, and gastric mucosal-associated lymphoid tissue lymphoma in human. In contrast, human pathogenic strains need adaptation to cause infection in mice, and mice infected with these adapted *H. pylori* exhibit mild infection. Gastric cancer is not induced even after long-term exposure without other stimuli or genetic defects, although the mouse may develop chronic atrophic gastritis [18], [19]. *H. pylori* can colonize the guinea-pig and the Mongolian gerbil and cause a severe inflammatory response but do not induce cancer in the absence of exogenous chemical carcinogens [20]. In contrast, rhesus monkeys have a mucin glycosylation similar to that of humans and naturally suffer from *H. pylori* infection, leading to loss of mucus, gastritis, gastric ulcers and even cancer, similar to what is found in human patients [21].

This variance in response to the pathogen colonization between different species signifies the necessity of suitable *in vitro* models as tools for investigating the mechanisms of human specific infections. *In vitro* microbial-mammalian co-cultures are used extensively to elucidate the mechanisms by which microbes adhere, invade and signal to the host, and to examine the mammalian cell response. However, human cell lines commonly used for *in vitro* infection studies have a highly variable expression of mucins and have very low production of gel forming-mucins [22]. Although shortened glycan chains may occur on some cell lines, many cell lines carry terminal sugars that are receptors for human pathogens, such as Lewis and sialyl-Lewis antigens [22], the host receptors for the *H. pylori* adhesins BabA and SabA. To mimic the gastrointestinal surface, a cell line needs to be able to form an adherent continuous polarized layer to ensure that bacteria interact with the apical surface of the cells, and to avoid introducing non-natural targets by adherence to the basolateral surface. Due to the use of non-polarized *in vitro* co-cultures, interactions that would not normally occur in humans unless the mucosa is injured or disrupted are often studied. In the present work the aim was to produce models suitable for investigating human/pathogen interactions with high similarity to the human gastrointestinal mucosal environment. Since models already exist that form adherent polarized epithelia (i.e. Caco-2 and MKN7), we aimed to enhance or induce the mucus layer of existing adherent polarizing cells, and in an alternative strategy, improve adherence and polarization of non-polarizing mucin producing cells.

We compared a range of treatments to enhance morphology, adherence, polarization and mucus production. To produce a polarized adherent layer we cultured the cells under air-liquid interface and semi-wet interface with mechanical stimulation. Furthermore, we compared the effects of galactose and glucose in the media, as replacement by galactose in previous studies indicated improvement in the formation of a tight epithelial cell layer [23], [24]. To induce mucin production, we used mechanical stimulation and additives to the media such as sodium butyrate (which has been shown to increase mucin mRNA [25], Prostaglandin E2, and *N*-[(3,5-Difluorophenyl)acetyl]-L-alanyl-2-phenyl]glycine-1,1-dimethylethyl ester (DAPT, (a Notch γ-secretase inhibitor that promotes goblet cell differentiation) [26]. In addition, we compared the effects of culturing the cells in RPMI1640 or DMEM, as DMEM has been reported to increase mucus production [27].

## Results

### Polarization of LS513 and MKN7 Cells in Air-liquid Interface Culture

**Figure 1:**
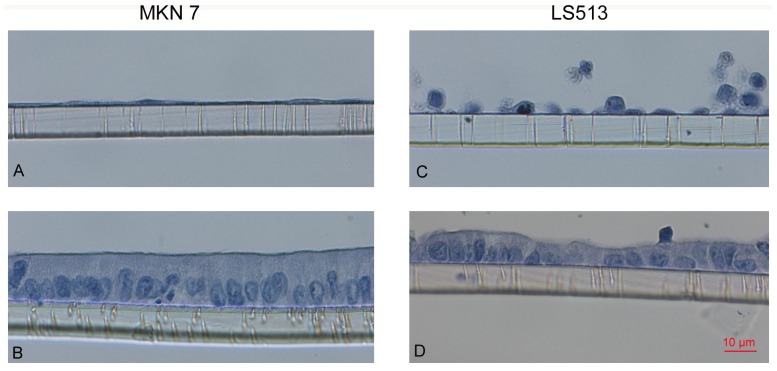
Culturing MKN7 and LS513 under air-liquid interface for 2 weeks resulted in adherent polarized monolayers. MKN7 (panels A and B) and LS513 (panels C and D) cultured on transwell membranes for 14 days post confluency in standard cell culture conditions (panels A and C) vs. air-liquid interface culture (panels B and D). The cells were seeded simultaneously at identical density and stained with hematoxylin.

We have previously demonstrated that the intestinal cell lines Caco-2, LS513 and PCAA/C11 and the gastric cell lines MKN1, MKN7, MKN28 and HFE-145 are capable of forming firmly adherent continuous cell layers, either spontaneously or after cultivation on special matrices, whereas other cell lines such as MKN45 and Kato III do not have this ability [22]. The cell lines also varied in mucin expression with MKN7 being most similar to gastric mucosa and Caco-2 and LS513 to the intestinal mucosa with regards to mucin expression, although none of them produced more than 1% of the amount of the mucin glycoproteins that build up the mucus layer in the *in vivo* gastrointestinal mucosa [22]. In spite of being the best cell line models, these models (as well as other *in vitro* models) still have morphologies vastly different from the *in vivo* morphology, with MKN7 growing as a very thin cell layer (Figure 1A) and LS513 as a disorganized layer (Figure 1B). To improve the morphology and mucus production, we cultivated the cells in air-liquid interface for two weeks post confluency. This treatment altered the cells to tall columnar cells with a basolateral nucleus similar to the appearance of epithelial surface cells *in vivo* (Figure 1C and D).

### Semi-wet Interface Culture and Mechanical Stimulation Improves Morphology and Mucus Production in LS513, HT29 MTX-P8 and HT29 MTX-E12 Cells

**Figure 2:**
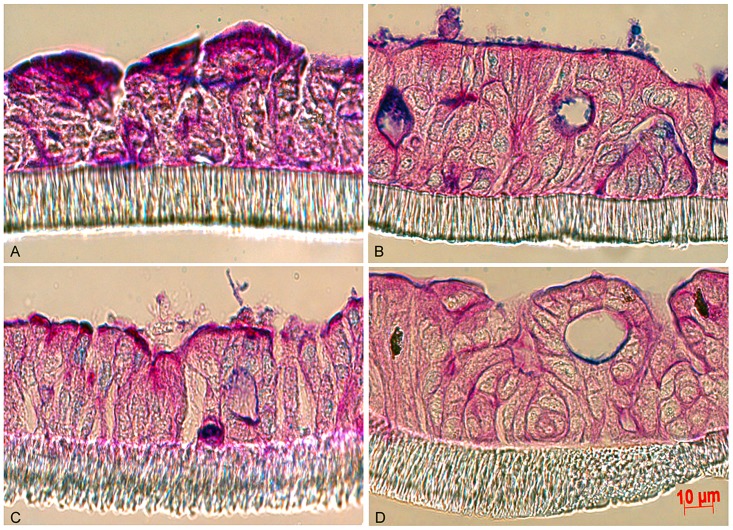
LS513 cells cultured for 3 weeks post confluency with different treatments. The LS513 intestinal cell line cultured under air-liquid interface in RPMI 1640 (panel A), treated with DAPT during the first six days of semi-wet interface with mechanical stimulation in RPMI 1640 (panel B), treated with 5 mM sodium butyrate and 10 µM DAPT while cultured under semi-wet interface with mechanical stimulation in RPMI 1640 (panel C) and treated with DAPT for the first six days in semi-wet with mechanical stimulation in DMEM (panel D). The cells had identical seeding density and were cultured for 21 days post confluency and then fixed in Carnoy’s methanol and stained with PAS/Alcian blue.

The morphology after air-liquid interface was not entirely homogeneous over the entire 1.1 cm^2^ area of the membrane, and it was still not possible to compel the cells to produce a firmly adherent mucus layer. Therefore, the cells were placed on a rocking board with a small volume of media in the apical compartment, as we hypothesized that the mechanical stimulation and continuous wetting of the apical surface would produce a more homogenous surface and stimulate mucus production, similar to how chondrocytes produce extracellular matrix after mechanical stimulation. Of the three different apical volumes tested (25 µl, 50µl and 100 µl), 50 µl gave the best result in LS513 cells (Figure 2A). The method of culturing the cells with 50µl in the apical compartment with continuous rocking is for the remainder of the manuscript referred to as “semi-wet interface with mechanical stimulation”.

To enhance the thickness of the mucus layer, *N*-[(3,5-Difluorophenyl)acetyl]-L-alanyl-2-phenyl]glycine-1,1-dimethylethyl ester (DAPT) was added to the basolateral side of cells cultured under semi-wet interface with mechanical stimulation. DAPT is a Notch γ-secretase inhibitor which promotes goblet cell differentiation. DAPT treatment during the first 6 days of semi-wet interface with mechanical stimulation culture had the best effect on production of mucus, and resulted in a thin adherent mucus layer on the apical surface (Figure 2B). Interestingly enough, the mechanical stimulation of LS513, either in combination with or without DAPT treatment, in addition to producing a thin adherent mucus layer, induced a three-dimensional architecture with shallow crypt like structures/lumen formation resembling the tissue architecture in the colon (Figure 2B vs. data not shown). DAPT treatment during the entire period of the semi-wet interface with mechanical stimulation or for the last 3 or 6 days did not produce as good results (data not shown).

Subsequently, we attempted to increase the mucus layer by adding potential mucin stimulators. Addition of sodium butyrate, which has previously been shown to increase mucin mRNA levels [25], did not enhance the thin mucus layer, but induced less crypt like structures in the cell layer (Figure 2C). Furthermore, it has previously been reported that DMEM increases mucus production [27]. However, we detected no increase in intensity of the mucus stain or in the thickness of the adherent mucus layer by culturing the cells in DMEM (Figure 2D) or by adding Prostaglandin E2 to the cells cultured with DAPT (data not shown).

Ussing chamber technology was used to measure the transepithelial potential difference (PD), membrane current (Im), Resistance (Rp) and capacitance (Cp). Transepithelial Im and PD can be used as a measure of membrane integrity (baseline value) and opening of ion channels (i.e. upon stimulus). Transepithelial resistance can be used as a measure of tight junction functionality (baseline value) and transfer of ions through the cell (upon stimulus, i.e. by opening of ion channels on both basolateral and apical membranes). Membrane capacitance is the ability of a membrane to hold an electrical charge and is proportional to its area. By measuring Cp, one can therefore get an indication of the surface area of the apical cell membrane. A cell with a flat surface area has a Cp of 1, whereas a cell with microvilli has a higher Cp. Furthermore, Cp can as a result be used to measure exocytosis upon stimulus due to the increase in membrane surface that occurs during this process [28].

LS513 cells cultured under standard conditions had an unstable and fluctuating PD and no Rp which lead to insufficient information for calculation other electrical parameters. In contrast, after the semi-wet interface with mechanical stimulation, the LS513 cells formed functional tight junctions with a resistance around 200 Ohm*cm^2^. The induction of increased mucin production by DAPT did not affect the Rp adversely (Table 1).

**Table 1:** Electrical parameters (mean±SEM) of LS513, HT29 MTX-P8 and HT29 MTX-E12 intestinal cell lines.

			PD (mV)	Im	Rp	Cp
				(mA/cm^2^)	(Ohm*cm^2^)	(mF/cm^2^)
**LS513**	**Standard conditions in RPMI media (n = 4)**		Fluctuating	No signal	No signal	No signal
	SWMS **conditions in RPMI media (N = 6)**		−0.6±0.10	−6.8±1.7*	211.7±32.6**	3.4±0.3**
	**DAPT treated SWMS conditions in RPMI media (n = 6)**		−1.1±0.42	−5.9±1.9*	207.7±53.1**	3.7±0.3**
	SWMS **conditions in DMEM media (n = 6)**		−0.36±0.42	−5.6±0.7*	131.4±8.7*	3.5±0.1**
**HT29 MTX-P8**	**Standard conditions in RPMI media (n = 4)**		−0.36±0.27	−0.4±0.4	148.4±42.1	2.2±0.2
	SWMS **conditions in RPMI media (n = 7)**		−1.56±0.28**	−1.8±0.2*	131.0±11.3	2.97±0.2**
	**DAPT treated SWMS conditions in RPMI media (n = 7)**		1.25±0.20**	−1.8±0.3*	123.8±8.6	3.0±0.2**
**HT29 MTX-E12**	**Standard conditions in RPMI media (n = 4)**		−0.03±0.03	−0.1±0.5	123.1±12.5	2.0±0.5
	SWMS **conditions in RPMI media (n = 7)**		0.43±0.05**	1.9±0.9**	155.9±11.5*	3.4±0.2*
	**DAPT treated SWMS conditions in RPMI media (n = 7)**		0.48±0.06**	2.2±0.5**	152.6±11.4	3.2±0.2*

Electrical parameters were measured in Ussing chambers for the LS513, HT29 MTX-P8 and HT29 MTX-E12 intestinal cell line cultured on snapwell membranes for 21 days post confluency. Under standard conditions the cells were immersed in media. Under semi-wet interface with mechanical stimulation conditions (SWMS), 50 µl of media was applied on the apical side whereas the basolateral side was immersed in media. 10 µM of DAPT was added basolaterally during the first 6 days of semi-wet interface to the DAPT treated cells. Data are presented as mean ± standard deviation.

* , **respectively indicates p<0.05 and p<0.01 compared to same cell line cultured under standard conditions, Anova, dunnetts post hoc.

**Figure 3:**
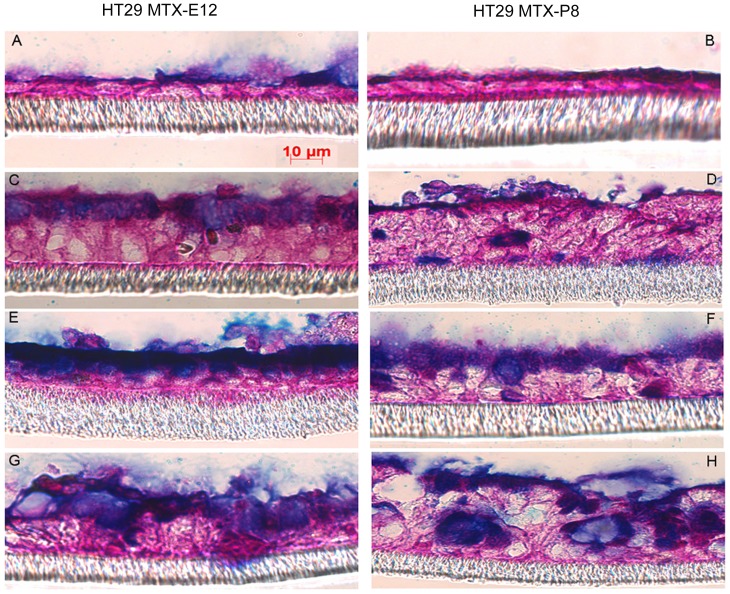
HT29 MTX-E12 and HT29 MTX-P8 cells cultured for 28 days post confluency with different treatments. The HT29 MTX-E12 (panels A, C, E and G) and HT29 MTX-P8 (panels B, D, F and H) intestinal cell line cultured under standard conditions in RPMI 1640 (panel A and B), under semi-wet interface with mechanical stimulation in RPMI 1640 (panel C and D), under semi-wet interface with mechanical stimulation treated with DAPT for the first six days in RPMI 1640 (panel E and F) and under semi-wet interface with mechanical stimulation treated with DAPT for the first six days in RPMI 1640 after stimulation by 1 mM carbachol in Ussing chambers (panel G and H). The cells had identical seeding density and were cultured for 28 days post confluency and then fixed in Methanolic Carnoy’s and stained with PAS/Alcian blue.

As semi-wet interface with mechanical stimulation and DAPT treatment had the most positive effect among the treatments used on LS513 cells, we attempted to improve the HT29 MTX-E12 and HT29 MTX-P8 cell lines which were previously reported to be adherent and produce a mucus layer [29], [30]. Culturing both cell lines in standard conditions for 28 days post confluency resulted in an adherent cell layer coated with a thin layer of mucus (Figure 3A and 3B). In the HT29 MTX-E12 the mucus layer was approximately 3 to 5 µm thick, whereas the thickness was less in the HT29 MTX-P8 cell line (Figure 3A and 3B). Semi-wet interface with mechanical stimulation altered their morphology to a three dimensional structure with some very shallow crypts/invaginations and increased the mucus layer thickness to 10–15 µm (Figure 3C and 3D). Stimulation with prostaglandin E2 produced no significant changes on these cell lines (data not shown). However, addition of DAPT during the first six days of semi-wet interface with mechanical stimulation increased the thickness of the mucus layer to 25–30 µm as measured by microscopy on fixed sections (Figure 3E and 3F). These measurements are likely to be an underestimate of the mucus layer thickness as it is difficult to retain the mucus layer during processing. This thickness resembles that of the adherent inner mucus layer of the *in vivo* intestinal surface, as the adherent layer in the ileum of the rat is around 30 µm [1] and that of the distal colon of mouse 49 µm [3].

**Figure 4:**
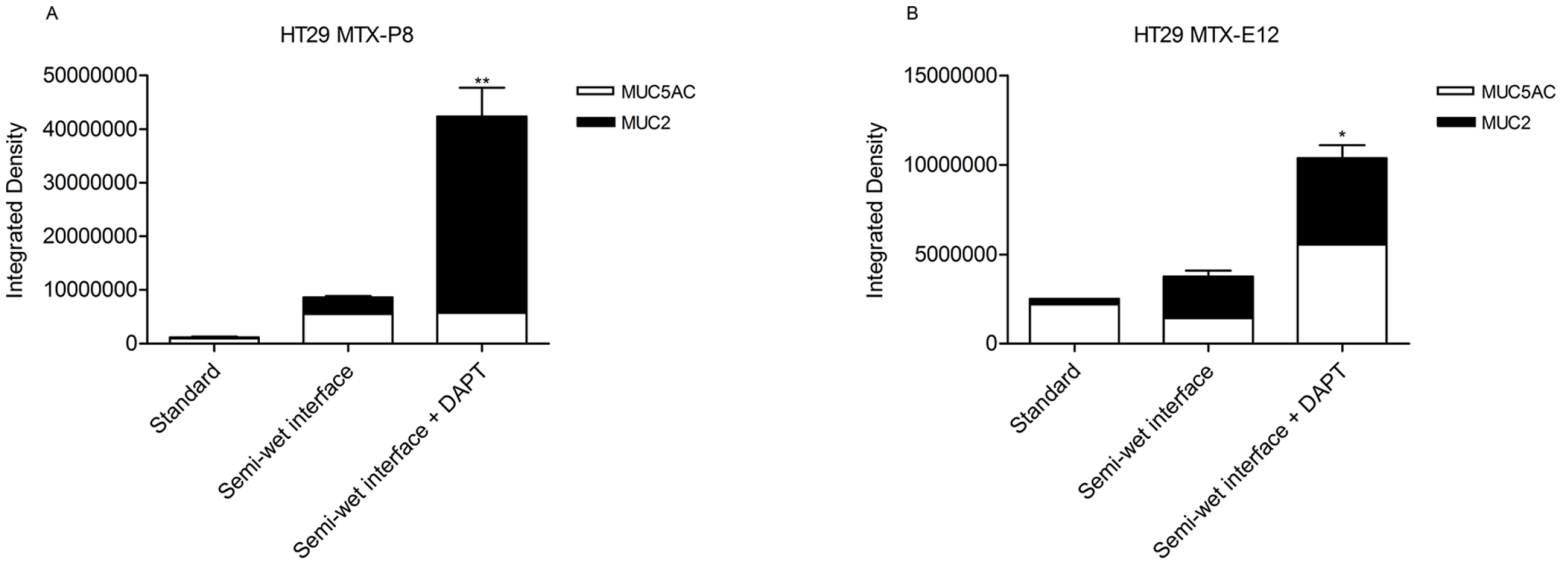
Quantification of MUC2 and MUC5AC in HT29 MTX cells cultured with different treatments. Comparison of the integrated density of fluorescence, measured by Image J software. The total amount of Muc2 and MUC5AC in HT29 MTX-E12 (A) and HT29 MTX-P8 (B) cultured with different methods. **P<0.01, *P<0.05 ANOVA, Dunnett’s post hoc, compared to control (n = 3).

**Figure 5:**
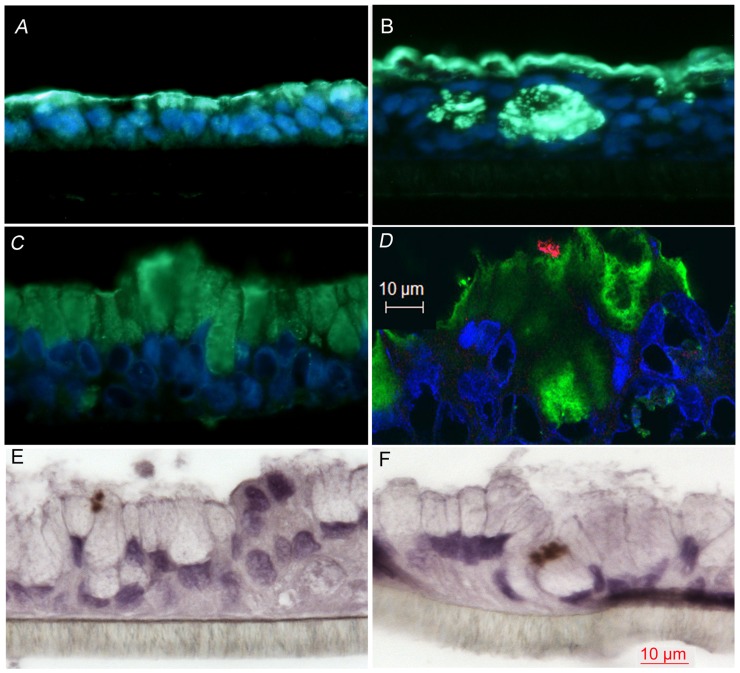
*C. jejuni* situ-hybridisation/MUC2 staining of HT29 MTX cell lines. Widefield fluorescence microscopy images of MUC2 staining (visualized as green) of HT29 MTX-P8 cells cultured in standard conditions (A), semi-wet interface with mechanical stimulation (B) and semi-wet interface with mechanical stimulation and DAPT treatment (C). Confocal microscopy images of in fluorescent in situ-hybridisation/MUC2 staining of HT29 MTX-E12 cultured in semi-wet interface with mechanical stimulation and DAPT treatment for 28 days after complete confluency and infected with *C.jejuni* for 24 h. MUC2 is visualized as green and *C. jejuni* as red (D). In situ-hybridisation using DAB as detection of *C.jejuni* (brown) with hematoxylin conterstain of HT29 MTX-E12 cultured in semi-wet interface with mechanical stimulation and DAPT treatment for 28 days after complete confluency and infected with *C.jejuni* for 24 h (E and F).

Analysis of HT29 MTX-E12 and HT29 MTX-P8 stained for MUC5AC and MUC2 with immunofluorescence further demonstrated a significant increase in mucin (MUC2+ MUC5AC, as these mucins are the most prominent gel forming mucins secreted by these cell lines) after culture in semi-wet interface with mechanical stimulation and DAPT treatment (quantification in Figure 4, images in Figure 5A–C). However, the immunofluorescence staining method results in loss of the mucus layer.

The transmembrane resistance of both HT29 MTX-E12 and HT29 MTX-P8 was around 150 ohm*cm^2^, which confirmed the ability of these cell lines to produce functional tight junctions under all tested culture conditions. The PD and Cp were considerably improved by semi-wet interface with mechanical stimulation with and without DAPT treatment (Table 1).

**Figure 6:**
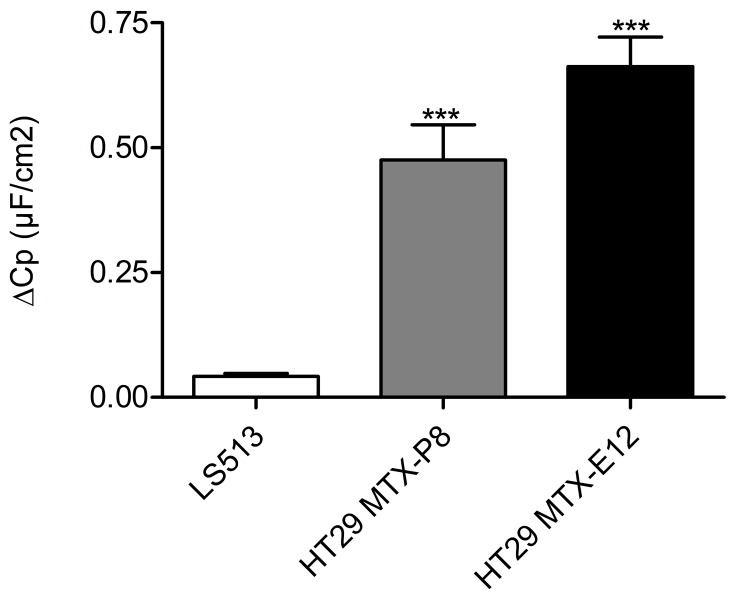
Transepithelial capacitance of HT29 MTX-P8 and HT29 MTX-E12 increases in response to carbachol. Changes in transepithelial capacitance (Cp) of LS513 (n = 6), HT29 MTX-P8 (n = 7) and HT29 MTX-E12 (n = 7) cell lines cultured in RPMI 1640 for 3 weeks under semi-wet interface with mechanical stimulation and DAPT treatment, measured by Ussing chamber after stimulation with cch (i.e.post cch Cp - baseline Cp values). ***P<0.001, Dunnett’s post hoc, compared to control.

The cholinergic agonist carbachol (cch) induces mucus secretion in the small and large intestine [31], [32]. Addition of cch to the basolateral side of the membrane resulted in mucin release from the goblet cells in to the mucus layer (Figure 3G and 3H). The mucus release in response to cch was further demonstrated by an increase of the capacitance of LS513, HT29 MTX-P8 and HT29 MTX-E12 cell lines, cultured in RPMI 1640 for 3 weeks under semi-wet interface with mechanical stimulation and DAPT treatment within 5 min after addition of cch. The increase in capacitance was significantly higher for HT29 MTX cells compared to LS513 cells (Figure 6). In contrast, cell lines with less mucin production, such as the MKN7 cell line did not respond to cch with an increase in Cp (data not shown). Thus, these cells have a response to mucin secretagogues similar to the *in vivo* intestinal mucosa.

Furthermore, as many mucosal pathogens, including *Campylobacter jejuni*, interact with mucus [7], [16], [33], we infected the *in vitro* model produced by culturing HT29 MTX-E12 cell line in semi-wet interface with mechanical stimulation and DAPT treatment with *Campylobacter jejuni* for 24 h under microaerobic conditions. Indeed, the results of fluorescence *in situ* hybridization with an eubacterial probe combined with MUC2 immunofluorescence staining demonstrated that the majority of the *C. jejuni* were located in the mucus layer in close contact with MUC2 (confocal image, Figure 5D), and some *C. jejuni* also attached to the epithelial surface (Figure 5E and F).

### Effect of Semi-wet Interface Culture and Mechanical Stimulation on Other Cell Lines

To investigate the ability of other gastrointestinal cell lines to produce a polarized adherent layer, we studied the electrical parameters and morphology of intestinal cells such as Caco-2, T84, HT29, LS174T, LS178TL8 as well as the gastric cells MKN7, MKN45, MKN1, AGS, NCI-N87 and its clones hTERT clone 5 and hTERT clone 6 cultured under semi-wet interface with mechanical stimulation. Among these, only the colonic cell lines Caco-2 (which is known to produce polarized monolayers and is used as an enterocyte model), T84 and the gastric cell line MKN7 produced a tight epithelium (Table 2). The LS178TL8 and MKN45 cell lines had a low epithelial resistance (below 100 Ohm*cm^2^), and the other cell lines had fluctuating PD and no Rp, confirming that the disorganized and un-polarized morphology resulted in inadequate ability to produce a tight layer. The Caco-2 cell line had highest Cp, reflecting that they have microvilli on their surface. In contrast, the cell line with the lowest Cp, MKN7, had a Cp of around 1 mF/cm, indicating that MKN7 has a relatively flat apical surface (Table 2).

**Table 2:** Electrical parameters (mean±SEM), measured in Ussing chambers for different gastrointestinal cell lines.

	Cell-line	PD (mV)	Im (mA/cm^2^)	Rp (Ohm*cm^2^)	Cp (mF/cm^2^)
**Intestinal**	Caco-2 (n = 8)	−2.68±0.66	−5.91±0.65	135.34±7.33	4.47±1.39
**cell lines**	T84 (n = 8)	−1.18±1.10	−4.7±9.20	284±189.2	1.85±0.38
	LS178TL8 (n = 6)	−0.2±0.034	−10.7±3.40	12.6±1.8	21.3±2.50
	LS174T (n = 8)	Fluctuating	No signal	No signal	No signal
	HT29 (n = 8)	Fluctuating	No signal	No signal	No signal
**Gastric**	MKN7 (n = 8)	−1.2±0.34	−1.3±0.20	248.7±35.95	1.08±0.28
**cell Lines**	MKN45 (n = 6)	−0.32	19.30	33.20	18.50
	MKN1 (n = 6)	Fluctuating	No signal	No signal	No signal
	AGS (n = 6)	Fluctuating	No signal	No signal	No signal
	NCI-N87 (n = 6)	Fluctuating	No signal	No signal	No signal
	NCI-N87 (hTERT-clone 5)(n = 6)	Fluctuating	No signal	No signal	No signal
	NCI-N87 (hTERT-clone 6)(n = 6)	Fluctuating	No signal	No signal	No signal

Cell lines were cultured on snapwell membranes for 28 days (for MKN7, HT29 MTX-P8 and HT29 MTX-E12) and 21 days (for the remaining cell lines) after confluency in semi-wet interface with mechanical stimulation.

The morphology of these cell lines were also examined using PAS/Alcian blue stained sections which confirmed inability of the cells with fluctuating PD or very low Rp (LS174T, HT29, MKN45, AGS or NCI-N87 and its clones 5 and 6) to produce an organized and adherent cell layer (data not shown). Unfortunately, these are also the cell lines that express the highest amount of the secreted types of mucins, whereas the cells with an adherent cell layer did not produce any major amounts of mucins of the secreted types as determined by Alcian blue/PAS staining.

### Mixed Cultures of Cells with Adherent Properties and Mucus Producing Properties did not Result in an Organized Polarized or Mucus Secreting Surface

Based on these results as well as a promising report about successful mixed culture of Caco-2 and HT29 resulting in a morphology resembling small intestine [34], we cocultured the intestinal Caco-2 cell line with the ability to produce a polarized layer with functional tight junctions with the mucin producing intestinal cell lines, HT29 and LS174T. Similarly, we mixed the tight junction forming gastric MKN7 cell line with the gastric mucin producing cell lines, MKN45 and AGS. Unfortunately, regardless of the different methods and concentration of the mixture (Table 3), the cells were not capable of producing an adherent monolayer or an ordered three-dimensional structure with a stable PD or Rp. Furthermore, the morphology of cells exhibited the scattered distribution of non-adherent (HT29/LS174 or MKN45/AGS) cells among the adherent cells (Caco-2 or MKN7) (data not shown). We also seeded the mucus producing cells on top of an adherent monolayer of Caco-2 or MKN7 cells, but this also failed to increase adherence or organization of the mucus producing cells. In addition to not being able to produce an organized, polarized mucosal surface with functional tight junctions, either individually or in combination with adherent cell lines, none of these mucus producing cell lines produced an adherent mucus layer (data not shown).

**Table 3:** Concentrations and methods used for mixing gastrointestinal cell lines.

Cell Lines	Con. of first cell line	Con. of second cell line	Comments
Caco-2+ HT29 or LS174	3×10^5^ Cell/well	3×10^5^ Cell/well	Mixture of cells was cultured at the same time.
Caco-2+ HT29 or LS174	5×10^5^ Cell/well	1×10^5^ Cell/well	Mixture of cells was cultured at the same time.
Caco-2+ HT29 or LS174	7.5×10^5^ Cell/well	5×10^5^ Cell/well	Caco-2 was cultured till complete confluency, then HT29/LS174 was added.
MKN7+ MKN45 or AGS	3×10^5^ Cell/well	3×10^5^ Cell/well	Mixture of cells was cultured at the same time.
MKN7+ MKN45 or AGS	5×10^5^ Cell/well	1×10^5^ Cell/well	Mixture of cells was cultured at the same time.
MKN7+ MKN45 or AGS	7.5×10^5^ Cell/well	5×10^5^ Cell/well	Caco-2 was cultured till complete confluency, then HT29/LS174 was added.

Gastrointestinal cell lines that produce an adherent layer on snapwell membranes (Caco-2 and MKN7) and cell lines with higher mucin production (HT29, LS174, AGS, MKN45) were cultured on snapwell membranes and kept in semi-wet interface for 21 days after confluency (n = 8).

**Figure 7:**
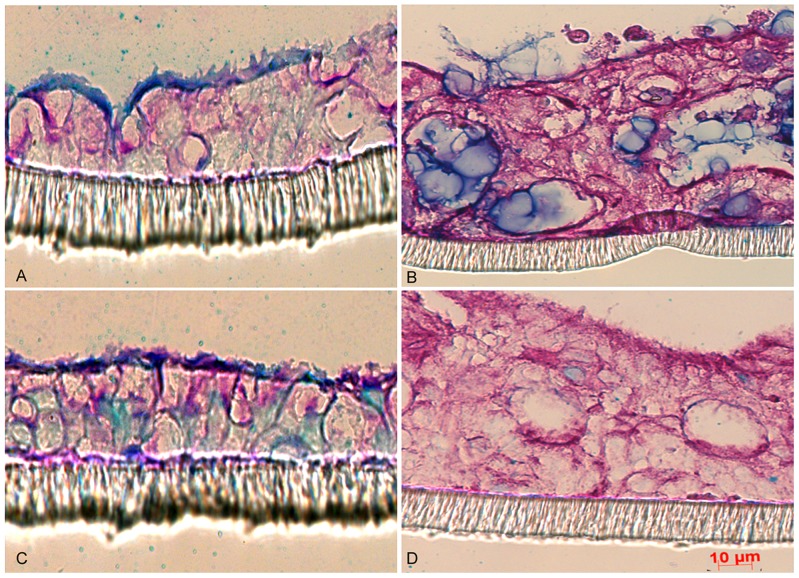
Caco-2 and T84 cultured under semi-wet interface with mechanical stimulation and DAPT treatment. Caco-2 (panel A and C) and T84 (panel B and D) intestinal cell lines treated with DAPT during the first six days of semi-wet interface with mechanical stimulation in RPMI 1640, stained with PAS/Alcian blue. These cell lines displayed an uneven morphology and mucin production in response to this treatment within each membrane; 10% of the membrane looked like A and B and 90% as panel C and D.

In an alternative strategy, to stimulate mucin production we treated the MKN7, Caco-2 and T84 (which were capable of producing a polarized cell layer with functional tight junctions) with DAPT during the first six days of semi-wet interface with mechanical stimulation (i.e. the same conditions that we used successfully on the LS513, HT29 MTX-E12 and HT29 MTX-P8 cells), which resulted in a mucin increase in the intestinal cells lines. However, these cells demonstrated variation in their ability to produce goblet cells within each membrane: small patches had a goblet cell density similar to the small intestine, but the majority of the membrane was devoid of goblet cells (Figure 7). Thus, they did not improve to the extent that rivaled the LS513, HT29 MTX-E12 and HT29 MTX-P8 cells, although the presence of these patches indicate that it might be possible to increase the goblet cell content of these models. There was no obvious improvement of the MKN7 cells after DAPT treatment (data not shown). Furthermore, addition of Prostaglandin E2 had no significant effect on these cell lines (data not shown).

These experiments demonstrated variation between different cell lines in response to our treatments and Table 4 provides a general overview of the effect of these methods on the gastrointestinal cell lines.

**Table 4:** Properties of cell lines after culture in semi-wet interface with mechanical stimulation.

Cell line	Polarized	3D-organisation	Mucus layer aftertreatment with DAPT	Mucus layer without treatment
NCI-N87 hTERT Clone 5	No	Disorganized	Loose/partial	Loose/partial
NCI-N87 hTERT Clone 6	No	Disorganized	Loose/partial	Loose/partial
AGS	No	Disorganized	No	No
MKN7	Yes	Monolayer	No	No
LS513	Yes	Shallow colonic crypts	Thin adherant layer	No
Caco-2	Yes	Very shallow crypts	Thin/partial layer	No
T84	Yes	Very shallow crypts	Thin/partial layer	No
HT29-MTX-P8	Yes	Multi-layer/Very shallow crypts	Thick adherant layer	Very Thin
HT29-MTX-E12	Yes	Multi-layer/Very shallow crypts	Thick adherant layer	Thin

For the mucus layer description, the cell lines with a “loose/partial” mucus layer, secretes mucus, which can be found in loose globs but not as an adherent mucus layer, the ones with a thin mucus layer corresponds to 5–10% of the in vivo thickness, and the ones with a thick mucus layer has a thickness similar to *in vivo*.

### The Cell Lines Varied in which Mucins they Expressed

**Figure 8:**
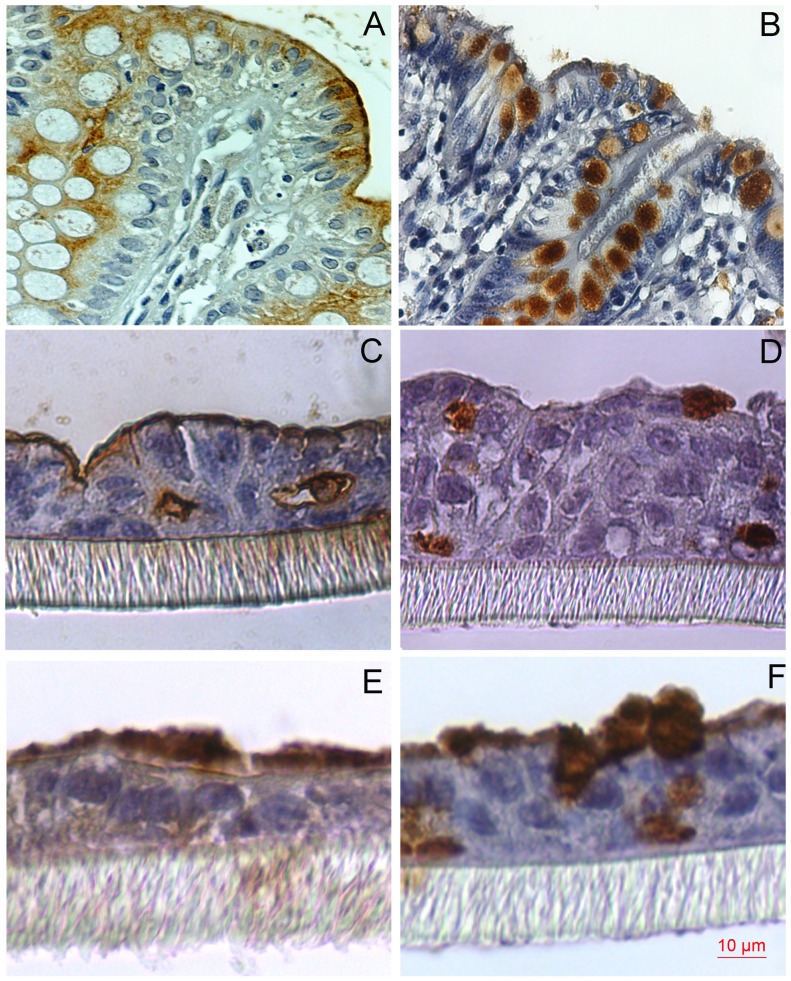
MUC13 and MUC2 Immunostaining. Immunohistostaining of human intestinal tissue (A), LS513 (C), and HT29 MTX-E12 (E) with the R20C1 antibody (recognizing MUC13), and intestinal tissue (B), LS513 (D) and HT29 MTX-E12 (F) with LUM2-3 antibody (recognizing MUC2), visualized in brown.

To determine if the cell lines have mainly gastric or intestinal mucin profiles and if the cells have a clear polarization with the membrane bound mucins located apically, we stained them for the gastric mucins MUC1, MUC5AC and MUC6 and for the intestinal mucins MUC2 and MUC13. MUC1 and MUC13 have a clear apical localization in normal healthy gastric vs. intestinal tissue [35], [36]. The MKN7, NCI-N87 hTERT clone 5 and 6, and AGS gastric cell lines had a high production of the gastric cell surface mucin MUC1. MUC5AC was detected in less than 30% of the cells of these cell lines and there was no trace of MUC6 production. A low percentage of the cells were also producing intestinal mucins MUC2 and MUC13 (Table 5). The HT29 MTX-P8, HT29 MTX-E12 and LS513 intestinal cell lines with the greatest resemblance to the human colon produced the secreted mucin MUC2 which was detected as an adherent layer on the apical surface of the cells as well as cell surface mucin MUC13 (Table 5 and Figure 8). However, MUC5AC and MUC1 were also produced by these cell lines, though in a diffuse manner. The proportion of cells expressing each mucin increased by 10–15% after DAPT treatment, but no changes were detected in response to PGE2 (Table 5). A more complete analysis of mucin expression has previously been performed on the MKN1, MKN7, MKN28, MKN45, HFE-145, LS513, Caco-2 and PCAA/C11 cell lines [22].

**Table 5:** Mucin expression of intestinal cell lines after culture in semi-wet interface with mechanical stimulation.

Cell line	MUC1	MUC2	MUC5AC	MUC6	MUC13
NCI-N87 hTERT Clone 5	90	2	25	0	5
NCI-N87 hTERT Clone 6	60	2	20	0	2
AGS	98	5	15	0	2
MKN7	100	0	20	0	10
LS513	5	40	80	0	70
HT29 MTX-E12	50	30	90	0	20
HT29 MTX-E12 (+DAPT)	70	50	95	5	40
HT29 MTX-E12 (+PGE2)	50	30	90	0	20
HT29 MTX-P8	50	50	80	5	50
HT29 MTX-P8 (+DAPT)	60	65	70	0	55
HT29 MTX-P8 (+ PGE2)	50	45	80	0	50

The number corresponds to the percentage of cells expressing the mucin as detected by immunohistochemistry.

### No Major Improvements were Achieved by Replacing RPMI with DMEM and Glucose with Galactose

Previous publications have indicated that mucus production increased using DMEM media [34], and that replacing glucose with galactose improves the formation of a tight epithelial cell layer [23], [24]. Therefore, the adherent, polarizing cell lines with low mucin production, such as LS513, T84 and MKN7 were cultured in DMEM media under semi-wet interface with mechanical stimulation. Thereafter, the capacity of cells to produce a polarized layer and their mucin production was studied by measuring the electrical parameters and staining with PAS/Alcian blue, respectively. LS513 cultured in DMEM had a lower resistance compared to cells grown in RPMI media, but still in the range that is acceptable for producing an adherent layer (Table 1). The morphology was almost the same with DMEM and RPMI media, and there were no evidence of increased mucin production (Figure 2D). MKN7 cells had higher resistance in DMEM media compared to the cells cultured in RPMI although there were no obvious changes in morphology of the cells, whereas for the T84 cell line resistance and morphology were the same in both media (data not shown).

**Figure 9:**
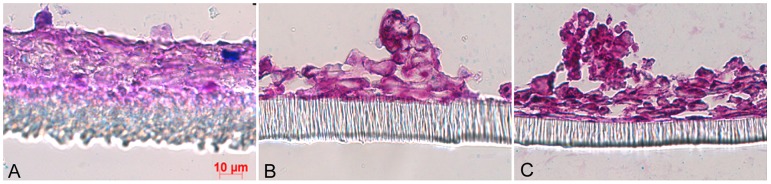
Gastrointestinal cell lines cultured under semi-wet interface in media containing 1% galactose. The intestinal cell line HT29 (panel A) and the gastric cell lines NCI-N87 hTERT clone 5 (panel B) and NCI-N87 hTERT clone 6 (panel C) cultured in glucose free RPMI 1640 with 1% galactose for 3 weeks under semi-wet interface with mechanical stimulation, stained with PAS/Alcian blue. These cells grew slower and more organized in galactose containing media than in glucose containing media, however, they did not achieve a highly organized and polarized *in vitro* gastric surface.

**Figure 10:**
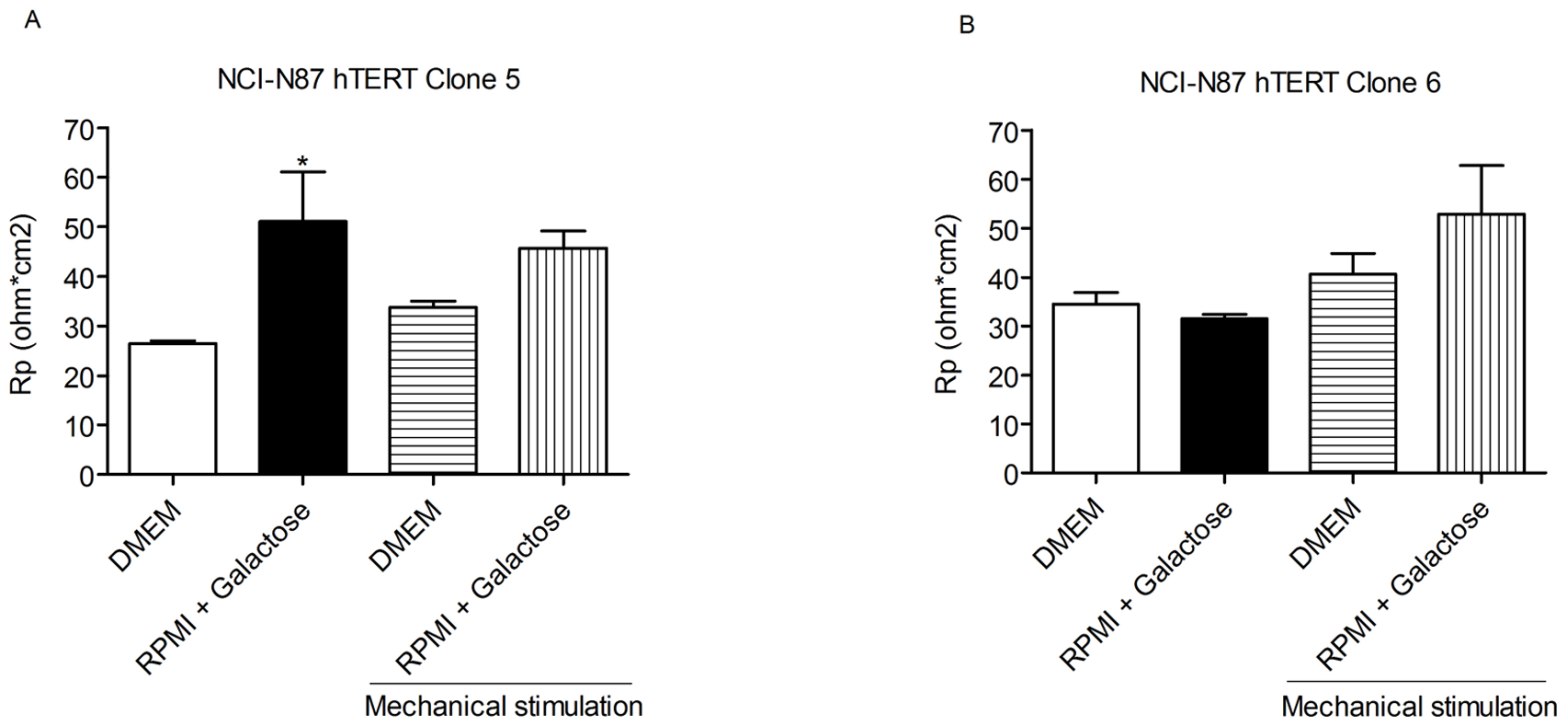
Transepithelial resistance of NCI-N87 hTERT clone 5 and clone 6 depends on culture conditions. Transepithelial resistance (Rp) of NCI-N87 hTERT clone 5 (panel A) and NCI-N87 hTERT clone 6 (panel B) cell lines cultured in DMEM or glucose free RPMI 1640 with 1% galactose for 3 weeks under semi-wet interface with or without mechanical stimulation, measured by Ussing chamber. *P<0.05 ANOVA, Dunnett’s post hoc, compared to control (n = 4).

Furthermore the mucus producing cell lines HT29, LS174T, AGS, MKN45, NCI-N87 (hTERT-clone 5) and NCI-N87 (hTERT-clone 6) which are incapable of producing an organized cell layer with functional tight junctions were cultured in glucose free media supplemented with galactose. HT29, NCI-N87 hTERT clone 5 and hTERT clone 6 showed slight changes in adherence and morphology (Figure 9 and 10). However, these changes did not improve the cell organization sufficiently to resemble an in vivo mucosal surface.

### Replacing the Media with FCS-free Media Replacement was Unsuccessful

Based on the increasing ethical concerns regarding the harvest of FCS from unborn calves and experimental problems caused by variations in different batches of FCS we attempted to replace the media with Ultra media (Lonza, Switzerland) on our most promising cell lines (HT29 MTX-P8 and HT29 MTX-E12). This media decreased the rate of cell growth in normal culture. Unfortunately, the media also completely reduced the ability of the cells to produce an adherent cell layer either under standard conditions, air-liquid interface, or semi-wet interface as well as had a negative impact on morphology and mucin production (data not shown).

## Discussion

The major goal of our study was to develop cell culture conditions that induce cell lines to resemble the gastrointestinal tract and thereby provide an *in vitro* mucosal surface suitable for host-pathogen interaction studies. Although gastrointestinal human cell lines exist that produce mucins or polarize, the models that reproducibly create the combination of a polarized epithelial cell layer, functional tight junctions and a thick adherent mucus layer have been missing until now. Here, we present a method, using standard laboratory equipment that can be used to alter the differentiation state and morphological organization of several cell lines so that they fulfill these requirements.

Firstly, using air-liquid and semi-wet interface culture, we could produce polarized cell layers with tall columnar cells and tight junctions, resembling the epithelial layer of mucosal surfaces from intestinal (HT29 MTX-E12, HT29 MTX-P8, LS513, Caco-2, and T84) and gastric (MKN7) cell lines. Air-liquid interface culture conditions have been reported to change the airway epithelial cells into polarized cells [37] and make the electrophysiological characteristics of rabbit conjunctival epithelial cells more akin those of the native tissue [38]. Thus, subjecting cell types that are in the air-liquid interface or semi-wet interface milieu *in vivo,* to these treatments *in vitro,* has the ability to make some of these cell lines more *in vivo*-like. This substantial improvement results in production of tight junctions which control paracellular permeability across epithelial cell sheets and also serve as a barrier to intramembranous diffusion of components between apical and basolateral membrane domains [39]. The polarization of the cell layer, which is not achieved using standard cell culture methods in many cell lines, is an important factor in producing immunity in response to invading pathogens. A study on intestinal epithelial cells using the T84 cell line showed that the polarized surface from which the antigen is internalized dramatically affects the functional outcome with regards to the generation of T-cell epitopes [40]. Furthermore, the polarity of epithelial cells is important for bacterial adhesion and invasion. For example, studies on *H. pylori* virulence demonstrated that CagA can disrupt the mechanisms maintaining cell polarity and adhesion to facilitate invasion of the mucosa [41]. These results indicate that using our *in vitro* models in comparison to standard cell culture methods of non-polarized cells would reduce the variation between the outcome of *in vivo* and *in vitro* experiments. Unfortunately, the semi-wet interface treatment is not effective for all gastrointestinal cell lines, especially not for some of the cell lines with a higher ability to produce mucin. The reason could be that in some of these cell lines the proteins important for the production of tight junctions are down regulated as a consequence of malignant transformation [39].

The second improvement in the semi-wet interface culture method in combination with DAPT treatment was the production of an adherent mucus layer. The presence of a mucus layer in an *in vitro* model of mucosa is necessary as mucins play an important role in the mucosal defense [7], [42]. The thickest mucus layer was produced by HT29 MTX-E12 and HT29 MTX-P8. HT29 MTX-E12 and HT29 MTX-P8 are (methotrexate selected) sub-clones of HT29, selected based on their goblet cells phenotype. Previous studies on different HT29 sub-clones indicate the ability of some of the subclones to secrete a mucus gel [30]. The HT29 CL16.E subclone appears to have more Alcian blue positive mucin compared with what we see in the HT29 E12 and E8 during standard culture [43]. Using SDS-PAGE/Western blotting, the same authors also demonstrated that at least the MUC2 fragments that are small enough to enter a 4%–8% polyacrylamide gel, increase when the cells are treated with forskolin and TPA [43]. Thus, combining the method developed herein with forskolin/TPA treatment on the HT29 CL16.E cell line, may give a model with a further enhanced mucus layer, although we did not investigate this as we did not have access to this subclone. However, we could not find any published information on the quantity of the different mucins produced by these other HT29 subclones, or on the thickness of the mucus produced by different sub clones. Using semi-wet interface in combination with DAPT treatment we achieved a substantial thick mucus layer covering HT29 MTX-P8 and HT29 MTX-E12 in comparison to the thin layer obtained during standard conditions. The thickness of mucus layer was increased to the same thickness as the mucus layer in the rat ileum [1] for the cells cultured in semi-wet interface with mechanical stimulation and DAPT treatment, whereas cells cultured in standard conditions had a mucus layer with approximately 1/10 of this thickness. In some experiments the mucus layer was thicker for HT29 MTX-P8, but HT29 MTX-E12 had a more reproducible morphology and mucin production during different experiments. Although the mucus layer in the LS513 cell line is only 5–10% of that present *in vivo*, it is still an improvement compared to other cell line models. The effect of DAPT on mucus production is in line with previous experiments demonstrating that γ-secretase inhibitors are involved in intestinal goblet cell metaplasia [26], and that goblet cell differentiation in mouse adenomas can be induced by blocking the Notch cascade with a γ-secretase inhibitor [44]. To increase the thickness of the adherent mucus layer, we tried other inducers of mucin production such as prostaglandin E2 and sodium butyrate, with or without DAPT treatment. However, we could not detect any changes in mucus protein production and thickness after any of these treatments. Furthermore, replacing RPMI media with DMEM, which previously has been shown to increase mucin production in HT29 clones [27], had no visible effect on the quantity of mucin production in our experiments. However, we detected a decrease in the epithelial resistance of the LS513 cell line when grown in the DMEM media. The negative effect of DMEM media on the integrity of the monolayer could be based on differences in glucose concentration between these media as the glucose concentration in the DMEM contains twice as much glucose as RPMI. A previous study demonstrated that replacement of normal media containing glucose with glucose free media could differentiate the HT29 cell line to form polarized cell layers with apical brush borders and tight junctions in a reversible manner [23]. Although we did not detect any such major differences, we found that most cell lines grew faster and in a more disorganized manner with higher glucose content compared to lower glucose content or glucose free media.

In response to CCh we detected mucin release from HT29 MTX-P8 and HT29 MTX-E12 in a manner similar to the intestinal goblet cells [31], indicating they are adequate models for *in vitro* studies on the stimulation of mucin in response to pathogenic invasion. These cells abundantly produced and secreted the major intestinal MUC2 mucin into the adherent mucus layer. Carnoy’s methanolic fixation is performed under water free conditions for optimal fixation of the mucus layer. Therefore, we aspirate the apical media, which would remove an outer loose mucus if such was present. We can thus not discern if the mucus layer is built up by two layers as in the murine colon [3], [45], or by a single layer as in the small intestine [1]. However, after infection we could detect the *C. jejuni* in the mucus layer, and a few attached to the epithelial surface, which is consistent with the Muc2 mucin forming a mucus barrier protecting the epithelial cells from the majority of bacteria.

We could also detect the gastric mucins MUC5AC and MUC1 in these cell lines, which is explained by that they are established from human colon adenocarcinomas, and cancer is known to induce changes in type of mucin production [46]. Another HT29 subclone, the HT29 16.E, has previously been shown to have shorter mucin glycans compared to mucin glycans from normal colon [47]. In our study, we have not analysed mucin glycans, however, it is quite possible that the mucin glycans on the cell lines that we present here also are not quite representative of the mucin glycans on the normal mucosal surface.

The T84 and Caco-2 cell lines also substantially changed morphology and increased the thickness of the adherent mucus layer after semi-wet interface culture with mechanical stimulation and DAPT treatment. However, this improvement was not consistent over the entire membrane but appeared in patches, indicating that these cell lines consist of different types of cells with different abilities to respond to these treatments. Therefore, it may be possible to pick clones from these cell lines that produce a more homogenous morphology and increased adherent mucus layer.

A third interesting feature of LS513, HT29 MTX-E12 and HT29 MTX-P8 cells cultured under semi-wet interface with mechanical stimulation conditions was the development of a three-dimensional architecture with shallow crypt like structures resembling the tissue architecture of the colon. The crypt like structures were more pronounced in the LS513 cell line than in HT29 MTX-E12 and HT29 MTX-P8.

In summary, we tested a range of culture methods on 14 cell lines with the aim to produce *in vitro* models that combine a polarized epithelial cell layer, functional tight junctions and an adherent mucus layer. The most dramatic effect on these parameters was the herein developed method of semi-wet interface culture in combination with mechanical stimulation and DAPT treatment. This method caused HT29 MTX-P8, HT29 MTX-E12, LS513 and, to some extent, Caco-2 and T84 cells to polarize, form functional tight junctions and to produce an adherent mucus layer.

## Materials and Methods

### Ethics

The human control samples used were collected after written informed consent according to the approval granted by the Human Research Ethics Committee, University of Gothenburg.

### Cell Types and Culture Conditions

Human intestinal cell lines Caco-2, LS513, HT29, T84 and LS174T (colorectal adenocarcinoma cells, ATCC), HT29 MTX-P8 and HT29 MTX-E12 (originated from HT29 and selected by growth adaptation to methotrexate, sequentially selected based on goblet cell morphology), as well as gastric cell lines MKN7, MKN45 (gastric adenocarcinoma cells, Riken Cell Bank, Japan), AGS (gastric adenocarcinoma cells, ATCC), NCI-N87 (gastric adenocarcinoma cells, ATCC) and its hTERT Clone 5 and hTERT clone 6 (clones from NCI-N87 transfected with human telomerase reverse transcriptase, kind gifts of Prof. M. Roxo Rosa, Universida Catolica Portuguesa) (Table 6) were cultured (at 37°C, 5% CO_2_, atmospheric O_2_) in a range of different media to analyze how this affected cell morphology: RPMI, DMEM/F12 (Lonza, Switzerland) containing 10% (v/v) FCS (Lonza, Switzerland) and 1% (v/v) penicillin-streptomycin (Lonza, Switzerland) as well as glucose free RPMI (Lonza, Switzerland) with 1% (w/v) galactose (AppliChem, Germany), 10% (v/v) FCS (Lonza, Switzerland) or FCS replacement media (Ultra media, Lonza, Switzerland) with 1% (v/v) penicillin-streptomycin (Lonza, Switzerland). Trypsin-versene solution (Lonza, Switzerland) was used for passaging the cells.

**Table 6:** Previously published characteristics of cell lines.

Cell line	Description
Caco-2	Origin: colorectal adenocarcinoma, Enterocyte like [52], produce MUC1, MUC3, MUC4, MUC5AC [53], MUC2 [54], MUC6 and MUC13 [22]
T84	Origin: colorectal adenocarcinoma, Goblet cell like, produce MUC1 and MUC2 [54]–[56]
LS174T	Origin: colorectal adenocarcinoma, Goblet cell like [57], produce MUC2 and MUC3 [54]
LS174T-L8	Origin: colorectal adenocarcinoma [58]
LS513	Origin: colorectal adenocarcinoma, produce MUC1, MUC2, MUC3, MUC5AC, MUC12 and MUC13 [22]
HT29	Origin: colorectal adenocarcinoma, heterogeneous (Goblet cells+enterocytes) [56]
HT29 MTX-12	Origin: colorectal adenocarcinoma, (originated from HT29 and selected by growth adaptation to methotrexate, selected based on goblet cells morphology) [59]
HT29 MTX-P8	Origin: colorectal adenocarcinoma, (originated from HT29 and selected by growth adaptation to methotrexate, selected based on goblet cells morphology) [60]
MKN7	Origin: gastric adenocarcinoma cells, produce MUC1, MUC4, MUC6 and MUC13 [22]
MKN45	Origin: gastric adenocarcinoma cells, produce MUC1, MUC5AC [61], MUC5B, MUC6, MUC13, MUC16 [22]
AGS	Origin: gastric adenocarcinoma cells [62], produce MUC5AC, MUC2 [63], MUC1 [64]
NCI-N87	Origin: gastric adenocarcinoma cells [65], produce MUC6 [66]
NCI-N87 hTERT clone5	Origin: NCI-N87 cells transfected with human Telomerase Reverse transcriptase
NCI-N87 TRET clone6	Origin: NCI-N87 cells transfected with human Telomerase Reverse transcriptase

The cells were grown on porous membranes (0.4 µm) with a 12 mm diameter providing a growth area of 1.12 cm^2^ (Snapwell™ insert, Corning, USA), supported by a detachable ring. All cultures were started by expanding the cells in flasks, then 7.5×10^4^ (with exception of 1×10^5^ for MKN7) cells were harvested and 200 µl of cells suspended in fresh media was added to the apical side of the membrane and 4 ml media to basolateral compartment. The standard condition was maintained by keeping this condition throughout the experiment. Air-liquid interface culture was performed with 2 ml media in the basolateral compartment and no media on the apical compartment after complete confluency. Semi-wet interface was produced by 2 ml media in the basolateral compartment and 25 µl, 50 µl, 75 µl or 100 µl media in the apical compartment after confluency. Mechanical stimulation and continuous wetting of the apical surface were achieved by placing the culture plates on a rocking board in the incubator. Basolateral media was refreshed every two to three days. For induction of mucin production 10 µM of DAPT (Sigma-Aldrich, Darmstadt Germany), or 5 mM sodium butyrate (Sigma-Aldrich, Darmstadt Germany), or 3 nM of Prostaglandin E2 (Sigma-Aldrich, Darmstadt Germany) was added basolateraly.

### Using Chamber Experiments

The snapwell tissue culture inserts were mounted in vertical Ussing chambers (exposed area 1.13 cm^2^). The basolateral side of the membrane was immersed in 115.8 mM NaCl, 1.3 mM CaCl_2_, 3.6 mM KCl, 1.4 mM KH_2_PO_4_, 23.1 mM NaHCO_3,_ 1.2 mM MgSO_4_ (KREB) solution containing 5.7 mM Na-Pyruvate, 5.1 mM Na-L-Glutamate 10 mM and D-Glucose, whereas the apical compartment was immersed in KREB solution containing 5.7 mM Na-Pyruvate, 5.13 mM Na-L-Glutamate and 10 mM and D-Mannitol. The solutions were gassed with 95% O_2_ and 5% CO_2_ at a temperature of 37°C and pH 7.4 throughout the whole experiment.

Transepithelial potential difference (PD) was measured once every minute during the whole experiment with a pair of matched Ag/Ag calomel electrodes (Radiometer, Copenhagen, Denmark) placed in saturated KCl and connected to the mucosal and serosal sides via a pair of 0.9%NaCl/6% agar bacteriological (Oxoid, England) bridges. After mounting the membrane in the Ussing chamber, PD was allowed to stabilize to achieve steady-state conditions. Epithelial resistance (Rp) and net membrane current (Im) were measured using square-pulse analysis: 5 V, 3 ms pulses were generated by a square pulse generator (Medimet, Gothenburg, Sweden) via a current limiting resistor (138 kΩ for intestinal cultures and 98 kΩ for gastric cultures) connected to a platinum electrode and applied across the membrane. The mean voltage response curve from twenty measurements was calculated and a linear fit was applied to the mean graph resulting in the voltage at time zero. Rp and Im were assessed in 4.5 min intervals for the entire experiment. Samples were stimulated with addition of 1 mM carbachol (Sigma-Aldrich, Darmstadt Germany) to the apical surface of the membrane. The basal values presented are the mean values pre stimulation. Delta values are used to illustrate effects induced by carbachol were calculated by subtracting the baseline value from the maximum effect.

### Fixation of Cells to Retain the Mucus Layer during Tissue Processing

A pair of membranes was cultured with the same conditions and before fixation one membrane was adjusted on top of the other membrane to avoid mucus washing away during processing. Methanolic Carnoy’s fixative (60% dried methanol, 30% chloroform, 10% acetic acid) was used overnight as the fixative.

### PAS/AB Staining

Formalin (4%) or Methanolic Carnoy’s fixed, paraffin-embedded cell lines cultured on Snapwell membrane (5 µm) were dewaxed and rehydrated. After rinsing in 3% acetic acid (Merk, Germany), the cells were stained using 1% Alcian blue (Merk, Germany) and oxidized in 1% periodic acid (Merk, Germany). The cells were then immersed in Schiff’s reagent (Sigma-Aldrich, Darmstadt Germany) and rinsed in 0.5% sodium meta-bisulphate (Merk, Germany). The measurement of mucus thickness was performed in the middle of the membrane using the ruler tool provided by the Zeiss Axio Observer/ApoTome.

### Mucin Immunostaining

Methanolic Carnoy’s fixed, paraffin-embedded cell lines cultured on Snapwell membranes (5 µm) were dewaxed and rehydrated. Antigen retrieval used was: 10 mM citric acid (Sigma-Aldrich, Dramstadt Germany), pH 6 at 99°C for 30 min then 40 min at room temperature followed by washing with PBS (0.15 mol/L NaCl, 5 mmol/L sodium phosphate buffer, pH 7.4) for MUC1, MUC5AC and MUC13, or with an additional step for the MUC2 and MUC6 antibodies using 10 mM 1,4-dithiothreitol (Fisher Scientific) in 0.1 M Tris/HCl buffer (pH 8.0) (37 degrees °C) for 30 min, followed by 25 mM iodoacetamide (Alfa Aesar, USA) at room temperature (in dark) for 30 min and washed with PBS. Sections were then treated with 3% (v/v) hydrogen peroxide (Fisher Scientific) for 10 min RT, washed twice with water and once with PBS containing 0.05% Tween 20. Nonspecific binding was blocked using serum free protein block (DAKO, Denmark) for 30 min and incubated with primary antibody for 1 h. Serum free antibody diluent (DAKO, Denmark) was used to prepare a 1∶4000 dilution of the 45M1 (recognizing MUC5AC, M5293, Sigma-Aldrich, Germany), LUM2-3 (recognizing MUC2 [48]), LUM6-3 (recognizing MUC6 [48]) antibodies whereas the R20C1 (recognizing MUC13 [49]) and BC2 (recognizing MUC1 [50]) antibodies were used at 1 µg/ml. The specimens were incubated with Broad Spectrum Zymed Poly HRP conjugated polymer (Invitrogen, USA) for 10 min followed by DAB chromogen (DAKO, Denmark) for 10 min and counterstained with haematoxylin for 1 min. The samples were washed 3 times with PBS containing 0.05% Tween-20 between each step and in distilled water after the final stain. A human sample from antrum was used as positive control for MUC1 and MUC5AC, duodenum for MUC6, small intestine for MUC2 and colon for MUC13 (Figure 8). The percentage of cells expressing the mucin was estimated using blinded scoring of the whole membrane.

### MUC5AC Immunofluorescence

The slides were deparaffinized, and antigen retrieval was performed by placing sections in 10 mM sodium citrate, pH 6.0 at 99°C for 30 min. After washing with PBS samples were blocked in protein block (DAKO, Carpinteria, CA). MUC5AC was detected using the 45M1 antibody (M5293, Sigma-Aldrich, Germany, 1/1000) for 1 h in RT and visualized by an anti-mouse Alexa Fluor 488 conjugate (1/100) (Invitrogen, USA), washed in PBS-T and mounted with Anti-fade containing DAPI (Invitrogen, USA).

### Infection with *C. jejuni*

Human intestinal cell lines HT29-MTX-E12 were cultured in RPMI (Lonza, Switzerland) containing 10% fetal calf serum (Lonza, Switzerland), 1% penicillin G sodium and streptomycin (Lonza, Switzerland). The cells were plated on snapwell tissue culture inserts (0.4 µm pores, 1.13 cm^2^ in diameter, Corning) at a density of 7.5×10^4^ cells/well and kept for 28 days after complete confluency in the semi-wet interface with mechanical stimulation to allow the cells to differentiate. During the first six days after complete confluency they were treated with 10 µM of DAPT (Sigma-Aldrich, Darmstadt Germany). For the infection experiment 7.1×10^7^ CFU of *C. jejuni* (clinical isolate) in 100 µl RPMI media was added to the apical side of the membrane and the culture was incubated under microaerobic conditions. 24 h after infection the membrane was fixed in Methanolic Carnoy’s.

### In situ Hybridization/MUC2 Immunofluorescence

The slides were deparaffinized, and then an abbreviated antigen retrieval step was performed by placing the slides in 10 mM sodium citrate, pH 6.0 at 95°C for 10 min. The slides were briefly rinsed in distilled water and air dried, and hybridization solution (40% (v/v) formamide, 20 mM Tris-HCl pH 7.4, 0.9 M NaCl, 0.1% SDS) containing 10 ng/µl of Cy3.5 5′ labeled eubacteria-specific probe (5′-GCTGCCTCCCGTAGGAGT-3′) [51] added, and incubated at 37°C in a humidified chamber containing 40% formamide overnight. The following day, the slides were washed with 0.9 M NaCl, 20 mM Tris-HCl pH 7.4 at 50°C for 20 min, followed by a brief submersion in room temperature distilled water, and then blocked at 4°C in serum-free Protein Block (Dako, Carpinteria, CA) for 1 h. The MUC2 immunohistochemistry was then carried out as per above, with all steps done at 4°C: the primary antibody, MUC2C3 (kind gift from G. Hansson, University of Gothenburg, Sweden) was diluted 1/1000 and incubated overnight, washed with PBS containing 0.05% Tween 20 (PBS-T), then an Alexa-Fluor 488 goat anti-rabbit secondary antibody applied overnight, washed in PBS-T and mounted with Anti-fade containing DAPI (Invitrogen, USA). A Zeiss LSM 510 META microscope was used for image acquisition and processing.

### In situ Hybridization for *C. jejuni*

The slides were deparaffinaized, and the endogenous peroxidase activity was inhibited by 3% H2O2 for 10 min, rinsed twice in distilled water and once with PBS containing 0.05% tween. The hybridization solution (40% (v/v) formamide, 20 mM Tris-HCl pH 7.4, 0.9 M NaCl, 0.1% SDS) containing 10 ng/µl of biotinylated eubacteria-specific probe (5′-GCTGCCTCCCGTAGGAGT-3′) [51] was added, and incubated at 37°C in a humidified chamber containing 40% formamide overnight. The following day, the slides were washed with 0.9 M NaCl, 20 mM Tris-HCl pH 7.4 at 50°C for 20 min, followed by a brief submersion in room temperature distilled water, and then blocked at 4°C in serum-free Protein Block (Dako, Carpinteria, CA) for 1 h. After that they were incubated with streptavidin (1 µg/ml) in antibody diluent for 2 h at 4°C, then rinsed with cold PBS containing 0.05% Tween and incubated for 10 min with DAB (Dako, Carpinteria, CA), rinsed in distilled water and counterstained with Hematoxylin.

### Integrated Density Analysis of MUC2 and MUC5AC in Immunofluorescence Images

A Nikon E1000 fluorescence microscope was used for image acquisition and processing. The quantification of integrated fluorescence density was obtained using the Image J program which is a public domain image processing program (Wayne Rasband at the Research Services Branch of the National Institute of Mental Health, National Institutes of Health, Maryland, USA). The full thickness of the layer on the membrane was outlined. The threshold for each stain was adjusted to the same level for all samples, and the same area was also analyzed for all samples. The measurement was then set for area and integrated density (the total intensity of fluorescence in the defined area).

### Statistical Analysis

The statistical analyses were performed using two-tailed T-test from SPSS software (IBM, USA) or ANOVA with Dunnett’s post hoc test (Graph Pad Prism, USA) on 4 to 8 samples per experiment.
